# Evaluation of Dietary Quality Based on Intelligent Ordering System and Chinese Healthy Eating Index in College Students from a Medical School in Shanghai, China

**DOI:** 10.3390/nu14051012

**Published:** 2022-02-27

**Authors:** Shaojie Liu, Jiangqi Wang, Gengsheng He, Bo Chen, Yingnan Jia

**Affiliations:** 1 Key Lab of Public Health Safety of the Ministry of Education, Department of Nutrition and Food Hygiene, School of Public Health, Fudan University, Shanghai 200032, China; liushaojie@fudan.edu.cn (S.L.); gshe@shmu.edu.cn (G.H.); 2 Key Lab of Public Health Safety of the Ministry of Education, Department of Preventive Medicine and Health Education, School of Public Health, Fudan University, Shanghai 200032, China; 18211020091@fudan.edu.cn; 3 Party and Government Office, Community Health Management Center, Kunshan 215301, China

**Keywords:** diet survey, investigative techniques, intelligent ordering system, Chinese healthy eating index, medical students

## Abstract

We intended to precisely evaluate the dietary quality of male and female medical college students using canteen data from the “Intelligent Ordering System” (IOS), combined with the supplemental food frequency questionnaire (SFFQ) and the Chinese Healthy Eating Index (CHEI) in Shanghai, China, to explore the potential factors influencing dietary quality. A total of 283 college students with the average age of 24.67 ± 3.21 years and Body Mass Index of 21.46 ± 3.49 kg/m^2^ in the medical school were enrolled in this study, and an online questionnaire investigation was conducted to collect the general information, lifestyle behavior, and SFFQ. The dietary data of the study participants from the school canteen were exported from the IOS of the Information Office of Fudan University. The CHEI consists of 17 components and the total score is 100, with a maximum score of each component of 5 or 10. We calculated each component score of the CHEI and aggregated the total score for male and female study participants. The Chi-square test and Wilcoxon rank sum test were employed in comparing the differences between the demographic characteristics and CHEI component scores of males and females. Univariate and multiple linear regression models were employed to examine the potential influencing factors of the total CHEI score. The CHEI median score was 66.65, and the component score for total grains was relatively low. Added sugars was the most overconsumed CHEI component. There were relatively serious deficiencies, based on the CHEI component scores, in fruits, soybeans, fish and seafood, and seeds and nuts in both sexes. Females had significantly higher CHEI scores than males (68.38 versus 64.31). The scores for tubers, total vegetables, dark vegetables, fruits, fish and seafood, dairy, and red meats were significantly higher in females than in males. Influencing factors including sex, education, dietary health literacy, and amount of time spent sedentarily were significantly associated with CHEI score. Our research revealed that the overall dietary quality needs to be further improved for college students at the medical school in Shanghai, China, with low intakes of total grains, fruits, soybeans, fish and seafood, and seeds and nuts, and high intakes of added sugars. Compared with males, females showed higher diet quality and conformed more strongly with the recommended Dietary Guideline for Chinese. Education, dietary health literacy, and amount of time spent sedentarily should be noted for improving the dietary quality of college students.

## 1. Introduction

The proportion of unhealthy dietary choices has increased dramatically in the past decades, representing an epidemic in many areas of the world. In the United States, suboptimal diet is considered the first leading cause of death and the third leading cause of death in disability-adjusted life years [1]. Mexico is undergoing a dietary shift toward unhealthy patterns, with increased intake of saturated fat, refined carbohydrates, and sodium [2]. In China, the traditional diet is also gradually changing to the unhealthy Western diet, including a decreased intake of coarse grains and an increased intake of oil and animal-sourced food [3]. Several studies report that unhealthy diet increases the risk of obesity [4], hypertension [5], cardiovascular disease [6], lung cancer [7], and metabolic syndrome [8].

Due to the transition of living and social environments from adolescence to young adulthood, combined with the development of individual independence and autonomy, young adults face continuous challenges to choose healthy food [9]. Stok et al. observed that dietary habits from adolescence to young adulthood usually become unhealthier because of individual, social, and environmental factors [10,11] such as the higher consumption of snacks and fried foods, and lower intake of fruits and vegetables [12,13]. Moreover, this transition may affect the establishment of long-term healthy dietary patterns, thereby affecting the risk of diet-related diseases [14]. At present, college students represent approximately half of the young adulthood population [15], and they are in a critical period of establishing healthy dietary patterns [16]. Therefore, it is essential to evaluate the dietary quality of college students so as to further improve their dietary habits.

Typically, the majority of college students still have dinner in fixed place such as school canteens. With the advent of digital technology, school canteens began to adopt novel technologies to modernize themselves [17], such as mixed deep learning and natural language processing technology. The primary characteristic of the modernized school canteen is the change in how dietary data are collected. Compared with the collection methods in traditional school canteens, including 3-day, 24-h diet recalls [18], food frequency questionnaires surveys [19], and weighed food records [20], the dietary data collection in modernized school canteens can be more accurate, automatic, and convenient, and resolve some limitations of traditional methods in terms of sample size, tracking time, and recall bias. The “Intelligent Ordering System” (IOS) was developed utilizing informational internet technology in the modernized school canteens in China, and it can realize multiple functions such as automatic dietary data collection, reservation order, dish management, and food safety monitoring in school canteens [21]. At present, the IOS has achieved a wide range of applications in the canteens of the medical school of Fudan University since September 2017 [21]. Our research based on the IOS can provide consecutive and relatively accurate meal data of each college student in the medical school canteens of Fudan University for diet quality analysis.

In addition to the consecutive and accurate dietary data, a suitable and comprehensive dietary assessment tool is also the basis for diet quality assessment in college students. There are a variety of dietary assessment tools built in different countries for evaluating dietary quality. Examples of these assessment tools include the Dietary Approaches to Stop Hypertension (DASH) score [22], the Mediterranean diet score [23], and the Healthy Eating Index [24]. Studies have been undertaken using the assessment tools of other countries to evaluate the dietary quality of the Chinese population; however, because the populations in different countries have different dietary habits and cultures, it is not appropriate to use dietary assessment tools of other countries in the Chinese population. The Chinese Healthy Eating Index (CHEI) was the first measurement tool to comprehensively evaluate the dietary quality of the Chinese population in accordance with the updated Dietary Guideline for Chinese (DGC-2016) and is suitable for Chinese adults, including college students [25]. The CHEI has been used to explore the association of dietary quality with primary liver cancer [26], breast cancer [27], and multi-ethnic colorectal cancer [28]. However, the dietary quality of Chinese college students has not been evaluated based on the CHEI score.

Dietary health literacy can influence dietary health behavior. Thus, dietary health literacy was regarded as a potential factor that might influence the dietary quality of college students in the medical school. Dietary health literacy was used to reflect the individual ability of acquiring, comprehending, and applying the dietary health information or services, and of making correct nutrition decisions [29]. Liao et al. indicated that dietary health literacy was associated with healthy-eating behavior, and thus, influenced the dietary quality in Taiwanese college students [30]. Similarly, Zoellner et al. found that the dietary health literacy of their study participants could effectively influence dietary quality [31]. Thus, dietary health literacy was considered in our research.

Our study aimed to (1) utilize dietary data from the IOS combined with the supplemental food frequency questionnaire (SFFQ) to relatively precisely evaluate dietary quality based on CHEI scores in male and female college students in a medical school in Shanghai, China, and (2) further explore the potential influencing factors of diet quality in college students at a medical school to provide a scientific reference for the subsequent improvement of students’ dietary habits.

## 2. Subjects and Methods

### 2.1. Target Population

**Figure 1:**
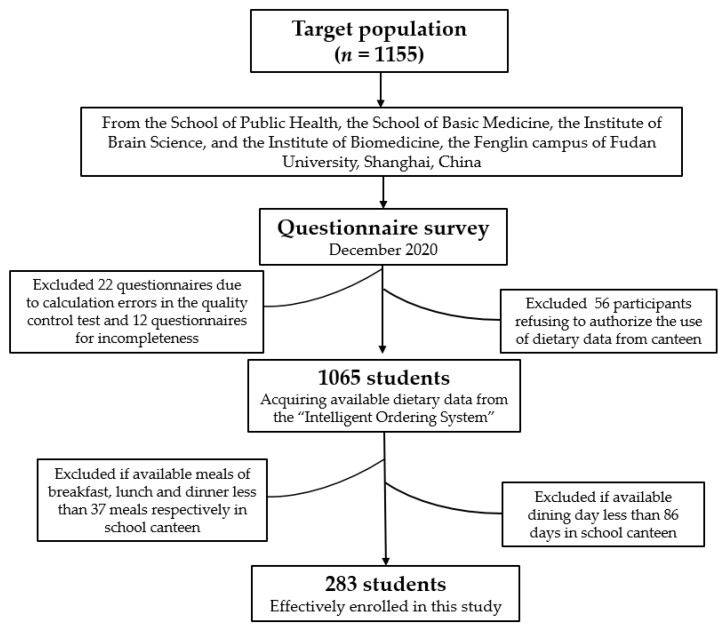
The flow chart of this study.

The sampling method was previously described [32], and random cluster sampling was applied. Four colleges and departments were randomly selected from the medical school of Fudan University, Shanghai, China, including the School of Public Health, the School of Basic Medicine, the Institute of Brain Science, and the Institute of Biomedicine. A total of 1155 college students from the medical school were invited to participate in our study, with the exception of first-year students who studied and lived on other campuses of Fudan University. The flow chart is shown in Figure 1, including the inclusion and exclusion criteria. Our study was conducted in accordance with the Declaration of Helsinki, and the protocol was approved by the Ethics Committee of Medical Research, School of Public Health, Fudan University (protocol code: IRB#2019-01-0726S; data: 7 January 2019).

### 2.2. Introduction of the “Intelligent Ordering System”

The IOS has been officially in use in the medical school of Fudan University since September 2017, and it is regarded as a convenient ordering and pricing tool for teachers and students to improve the environmental level and dining efficiency of the medical school canteens. Moreover, the IOS can obtain long-term, relatively accurate records of each meal of both teachers and students in the medical school canteens. The specific ordering process is as follows. The backend of the IOS inputs detailed common data from dishes, and the merchant will display the corresponding dishes on the system according to the dishes provided by the school canteens that day. The large screen of the system displays a picture of the dish, as well as the name and the price of the dish for teachers and students to view (Figure S1). After students and teachers select the dishes and check the price, each meal’s information will be automatically recorded in the restaurant’s ordering terminal and organized into the continuous dining data. The reliability and validity of the system were verified in a similar system in Australia [33].

### 2.3. Acquiring the Available Dietary Data from the “Intelligent Ordering System”

A total of 1065 college students at the medical school agreed to have their dietary data exported and used by the Information Office of Fudan University of Shanghai, China, from September 2018 to September 2019. The dietary data of study participants were obtained from the IOS, including meal dates and times, the names of the selected dishes, and the numbers of servings. Subsequently, we converted all meals to grams of food and condiments based on the daily intake for each student. We then weighed the total raw materials, each condiment (such as oil, sodium, and sugar), and cooked dish for each plate of food from the back kitchen of the students’ canteen. Next, each portion of food was weighed five times at the canteen’s window, and the average number of grams was taken into consideration. For example, we needed to calculate each intake of each serving of “fried rice with egg” supplied by the school canteen. First, all raw materials (rice, egg, condiments) were weighed before cooking. Second, cooked “fried rice with egg” dishes were weighed after cooking in the back kitchen of the school canteen. Third, we ordered and weighed each serving of “fried rice with egg” five times at the order window. The average value of “fried rice with egg”, measured in grams, was calculated and applied for each serving given to the consumer each time they visited the school canteen. Finally, the raw food materials (rice, egg, condiments) of each serving of “fried rice with egg” could be calculated depending on the ratio of the grams of cooked product for each serving. The total energy (kcal/d) and the grams of food and condiments were calculated, with the combined weighted data from the canteens and the meal data exported from the IOS with the Chinese Food Composition (version 2019). Based on the meal data from the IOS and food composition of each meal in one year, we could calculate the average intake of each food and condiment per day for study participants dining in the medical school canteens. Then, all foods were converted into food groups according to the components of the CHEI, such as total grains, fruits, and dairy. In order to guarantee the accuracy of dietary evaluation in school canteens, the study participants needed to eat in the school canteens on more than 1/3 of school days, except for holidays. Thus, the participants were excluded if they dined in the school canteen on less than 86 days, with less than 37 meals of breakfast, lunch, and dinner. In total, 283 subjects were effectively enrolled in our study. During the school day of one year, 283 subjects ate in the school canteens on 142 days on average, consuming a total of 426 meals, including 117 meals for breakfast, 170 meals for lunch, and 139 meals for dinner, respectively.

### 2.4. Questionnaire Survey

In December 2020, we conducted an online questionnaire survey for our target population. The survey method was previously described [32]. The online questionnaire survey was conducted via “Wenjuanxing” (https://www.wjx.cn/app/survey.aspx, accessed on 12 December 2020), a platform providing functions equivalent to Amazon Mechanical Turk. General information (age, sex, education, major, household type, resident/non-resident, height, and weight), lifestyle behavior (smoking status, dietary habits, Nutrition Literacy Assessment Questionnaire, amount of time spent sedentarily, and leftovers rate), SFFQ (food items such as dairy, fruits, nuts, beverages, and alcohol, are not provided or under-provided in the medical school canteen), and the changing situation of dietary habits in the past two years were collected. Additionally, we acquired the authorization to use students’ dietary data provided by the IOS from the Information Office of the medical school of Fudan University of Shanghai, China, from September 2018 to September 2019 [21]. Two simple mathematical calculations, “what’s 5 plus 9?” and “what’s 8 plus 9?”, were designed for quality control of the questionnaire, with 22 participants and 12 participants excluded due to calculation errors in the quality control test and for incomplete questionnaires, respectively. Informed consent forms were also included in the questionnaire, and 56 participants refused to authorize the use of dietary data from the IOS. In total, 1065 college students at the medical school successfully finished the questionnaire survey.

### 2.5. CHEI Calculation

The CHEI was built on the latest Dietary Guidelines of Chinese (DGC-2016), which has the capacity to identify the differences in dietary quality among Chinese people. The total CHEI score is 100, which consists of 17 components (with a maximum score of each component of 5 or 10) [25]. Moreover, the reliability and validity of CHEI was previously validated [34], and it is widely applied around the world. The higher the CHEI score, the more in line the diet with the recommendations of DGC-2016, including higher consumption of total grains, whole grains and mixed beans, tubers, total vegetables, dark vegetables, fruits, dairy, soybeans, fish and seafood, poultry, eggs, and seeds and nuts, and lower consumption of red meats, cooking oils, sodium, added sugar, and alcohol. For each food group of the SFFQ, two questions were asked to estimate the intake frequency and the amount per intake. We could evaluate the intake grams of each food group per day for study participants depending on the SFFQ. Finally, the dietary data of SFFQ were converted to intake grams of each food group per day according to the components of the CHEI, and integrated into the dietary database from the IOS. The leftovers rate of each participant was considered into the calculation of daily food intake. We calculated each CHEI component score and aggregated the total score for study participants based on the dietary database using the calculation formula of the CHEI.

### 2.6. Assessment of Covariates 

To identify potential factors of dietary quality, we collected the Nutrition Literacy Assessment Questionnaire, which was used to effectively evaluate the dietary health literacy of Chinese college students. With a total score of 65, the Nutrition Literacy Assessment Questionnaire was developed by Wang [35] based on the Critical Nutrition Literacy of Guttersrud [36] and the Assessment Indicators System of Health Literacy of Zhang [37], and consists of 13 items (Table S1) [35], which were divided into three dimensions: Acquisition Capacity (score ty20), including questions such as: “Do I discuss food with others?” and “Do I often refer to the information in the media?”; Comprehension Capacity (score ty30), including questions such as: “Do I understand the concept of ‘balanced diet’?”; and Application Capacity (score yi15), including questions such as: “Am I willing to spend extra time or money on healthy meals?”. The reliability and validity of the Nutrition Literacy Assessment Questionnaire was previously validated [35].

Age was sorted as less than 26 years old and greater than or equal to 26 years of age, with education level sorted as undergraduate and graduate. The following dichotomous variables included sex (male, female), major (medical major, medical-related major), household type (urban, countryside), resident student (yes, no) and dietary habit (general diet, other diet such as Muslim and vegetarian diet). The amount of time spent sedentarily was evaluated by sitting time (hour/day). The leftovers rate was evaluated by asking the average leftovers rate (%) of each meal consumed in the medical school canteens for each student in the questionnaire. Body Mass Index (BMI, kg/m^2^), determined as weight (kg)/height^2^ (m^2^), was divided into three levels according to the Working Group on Obesity in China [38], including underweight (<18.5 kg/m^2^), normal weight (18.5–23.9 kg/m^2^), and overweight and obesity (≥24 kg/m^2^). Smoking was defined as at least one cigarette a day for more than six months.

### 2.7. Statistical Analysis

Continuous variables were described as median (inter-quartile range, IQR) and categorical variables were described as frequency (ratio). The Chi-square test and Wilcoxon rank sum test were applied in an equilibrium test between demographic characteristics of males and females. The Wilcoxon rank sum test was used to compare the differences of the CHEI and its component scores in male and female participants. We converted the continuous variables corresponding to the CHEI component scores into four categorical variables, using the cutoff values of 0, 2.5, and 5 for all components with the exception of fruits, sodium, and cooking oils (the four intervals were score = 0, 0 < score < 2.5, 2.5 ≤ score < 5, and score = 5), and 0, 5, and 10 for fruits, sodium, and cooking oils (the four intervals were score = 0, 0 < score < 5, 5 ≤ score < 10, and score = 10). The differences in the four categories of the 17 CHEI components between males and females were measured by Chi-square test. Sex, education, household type, major, resident/non-resident student status, dietary habits, Nutrition Literacy Assessment Questionnaire, BMI, smoking status, and amount of time spent sedentarily were all used as independent variables, and the total CHEI score was used as the dependent variable. The variable of the total CHEI score obeyed the normal distribution, and univariate and multiple linear regression models were applied to examine the potential influencing factors of the total CHEI score by calculating the *β* and corresponding 95% confidence intervals (95% CI). All statistical analyses were conducted using SAS software version 9.4 (SAS Institute Inc., Cary, NC, USA), with two-sided *p* values < 0.05 being considered statistically significant.

## 3. Results

The demographic characteristics of 283 college students in the medical school, with an average age of 24.67 ± 3.21 years and a BMI of 21.46 ± 3.49 kg/m^2^, stratified by sex, are displayed in Table 1, with significant differences in education, dietary habits, smoking status, Nutrition Literacy Assessment Questionnaire, and leftovers rate according to sex (*p* < 0.05). The mean value of sedentary time was 8.29 ± 5.01 h/day for 283 study participants. The percentages of participants who smoked (3.60%), were overweight or obese (31.5%), and followed other diets such as Muslim or vegetarian diets (9.00%) were found to be significantly higher in male participants than female participants. Additionally, the percentages of graduate students (64.00%) and underweight participants (21.50%) were significantly higher in females compared to males. The mean values of the acquisition, comprehension, and application capacity of the Nutrition Literacy Assessment Questionnaire were 11.78 ± 3.50, 22.15 ± 3.90, and 10.73 ± 2.47 for 283 study participants, respectively. Female participants were significantly higher than male participants in terms of acquisition, as well as in their application capacity for the Nutrition Literacy Assessment Questionnaire (*p* < 0.001). Moreover, the mean value of the leftovers rate was 13.18 ± 11.37% for 283 study participants, and the leftovers rate of females was also significantly higher than males (*p* < 0.001).

Table 2 showcases the CHEI and its component scores for college students in the medical school with a median score of 66.65 and compares the differences between males and females. The component scores for total grains, fruits, soybeans, fish and seafood, and seeds and nuts were relatively low. A comparison of the CHEI and its component scores between males and females revealed that females exhibited significantly higher CHEI scores than males (68.38 versus 64.31, *p* < 0.001). Further, females exhibited significantly higher scores for tubers, total vegetables, dark vegetables, fruits, fish and seafood, dairy, and red meats compared to males (*p* < 0.05). In contrast, the score for total grains was significantly higher in males than in females (3.25 versus 2.78). The results indicate that there are marked differences between males and females in the CHEI and its component scores.

**Figure 2:**
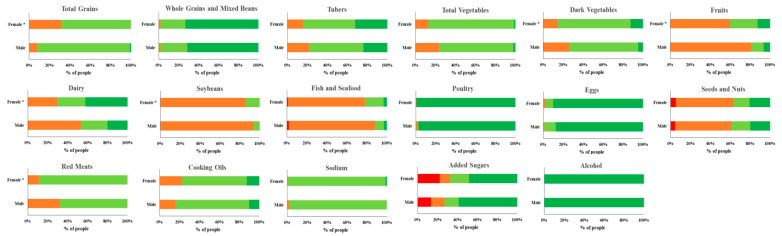
Proportion of four categories of CHEI component score for 283 college students in the medical school stratified by sex. For all components except for fruits, sodium, and cooking oils, the four categories are score = 0 ■, 0 < score < 2.5 ■, 2.5 ≤ score < 5 ■, score = 5 ■. For fruits, sodium, and cooking oils, the four categories are score = 0 ■, 0 < score < 5 ■, 5 ≤ score < 10 ■, score = 10 ■. * Significant differences in four categories of 17 CHEI components between males and females (χ^2^ test), *p* < 0.05.

We further converted the continuous variables corresponding to CHEI component scores into four categorical variables, which used the cutoff values of 0, 2.5, and 5 for all components with the exception of fruits, sodium, and cooking oils (the four intervals were score = 0, 0 < score < 2.5, 2.5 ≤ score < 5, and score = 5), and the cutoff values of 0, 5, and 10 for fruits, sodium, and cooking oils (the four intervals were score = 0, 0 < score < 5, 5 ≤ score < 10, and score = 10). Figure 2 showcases the proportions of the four categories of the CHEI component scores for 283 college students in the medical school, stratified by sex. In summary, with the exception of poultry, eggs, whole grains and mixed beans, and alcohol (more than 50% of students obtained the maximum scores for CHEI components), the majority of both male and female students were unable to follow the recommendations of DGC-2016. There were relatively serious deficiencies for CHEI component scores for fruits, soybeans, fish and seafood, and seeds and nuts in both males and females (with a CHEI component score of less than 2.5 or 5 in more than 50% of students). Added sugars were the most overconsumed CHEI component in both sexes. Moreover, the proportions of students who acquired zero points for the CHEI components of fish and seafoods, seeds and nuts, and added sugars were 1.80%, 4.50%, and 13.51% for male students, and 1.16%, 5.23%, and 22.09% for female students, respectively. There were significant differences for four categories of 17 CHEI components between males and females for total grains, dark vegetables, fruits, dairy, soybeans, and red meats. Compared with males, females exhibited higher scores for dark vegetables, fruits, dairy, soybeans, and red meats, while males scored higher than females for total grains (*p* < 0.05).

Table 3 showcases the factors potentially influencing the CHEI score. In the univariate linear regression model, female and graduate students were positively associated with the CHEI scores, but the overweight and obesity group were negatively associated with the CHEI scores (*p* < 0.05). The acquisition and application capacity of the Nutrition Literacy Assessment Questionnaire were positively associated with the CHEI scores, but time spent sedentarily was negatively associated. After all variables were introduced, sex and education displayed similar results, as yielded by the univariate linear regression analysis, with *β* (95% CI) values of 2.80 (1.24, 4.35) and 1.56 (0.23, 2.89), respectively. The application capacity on the Nutrition Literacy Assessment Questionnaire was positively associated with the CHEI score, with a *β* (95% CI) value of 0.34 (0.03, 0.66). The sedentary time was negatively associated with the CHEI score, with a *β* (95% CI) value of −0.16 (−0.28, −0.03).

## 4. Discussion

In our study, we utilized the continuous, one-year person-meal dietary data from the IOS combined with SFFQ to relatively precisely evaluate the dietary quality based on CHEI scores in 283 male and female college students in a medical school in Shanghai, China. This was the first accurate calculation of respondents’ daily intake over a prolonged period of time based on the IOS combined with the SFFQ to reveal the dietary quality of 283 participants based on CHEI score, to identify greater and lesser consumed foods in college students at the medical school in relation to the DGC-2016. The results indicated that the overall dietary quality of participants needs to be further improved. Most students were unable to follow the recommendations of the DGC-2016 with the exception of poultry, eggs, whole grains and mixed beans, and alcohol. The component score for total grains was relatively low. Fruits, soybeans, fish and seafood, and seeds and nuts were significantly deficient in both sexes. Females demonstrated greater dietary quality than males, and had higher CHEI component scores than males for tubers, total vegetables, dark vegetables, fruits, fish and seafood, dairy, and red meats, while the component score for total grains was higher in males than in females. After adjusting for all covariates, females and graduate students were positively associated with the CHEI scores. The application capacity of the Nutrition Literacy Assessment Questionnaire was positively associated with CHEI scores, and the amount of time participants were sedentary showed the opposite association.

Our findings extended the previous evidence based on a cross-sectional survey that dietary quality was relatively poor in college students. The dietary quality of 283 college students in the medical school was evaluated by CHEI, and the median score was just over 60 (with a maximum score of 100). From a CHEI component score standpoint, the score for total grains was relatively low, and fruits, soybeans, fish and seafood, and seeds and nuts were significantly deficient in college students of both sexes. This outcome is consistent with research from the United States [39] and Europe [40], which demonstrates that university students exhibit a high consumption of fried foods and high fat food, and a low consumption of fruits and vegetables from the canteen. This finding may be related to college students’ lack of nutritional knowledge. Werner et al. [41] determined that no more than 23.9% of college students answered questions regarding specific nutritional recommendations correctly, with even medical students exhibiting multiple deficiencies in nutritional knowledge [42]. Our study’s participants were also college students from a medical school and verified the above viewpoint. However, efforts need to be undertaken to further improve nutritional knowledge through social, family, and school environments in college students at the medical school. Not only is a healthier diet in school needed, but also the joint efforts of society, such as the Front-of-Pack initiative, an internationally advocated dietary label [43].

Moreover, we observed that the consumption of fish and seafood was seriously insufficient in study participants. A total of 1.80% of male students and 1.16% of female students acquired zero points for the CHEI components of fish and seafood, with this phenomenon also noted in several studies globally. Papadaki et al. showed that undergraduate students decreased their weekly consumption of oily fish and seafood since starting university and living away from home [44]. AlJohani et al. also observed that health sciences university students exhibited insufficient seafood consumption [45]. This may be related to the types of dishes provided by school canteens. Based on the menus provided for one year in this study, meat-containing dishes accounted for 20.20% of the total 886 dishes; however, fish and seafood-containing dishes accounted for only 5.98%, demonstrating that the canteen did not provide sufficient fish and seafood to satisfy the needs of students. A possible explanation is that fish and seafood are susceptible to rancidity, lipid oxidation, and microbial deterioration [46], and as such, school canteens decrease the supply of fish and seafood to prevent the occurrence of food safety issues. However, fish and seafood are rich sources of poly-unsaturated fatty acids, and play an important role in protecting cardiovascular health [47]. Thus, school canteens need to provide students with sufficient fish and seafood on the premise of ensuring food safety based on the recommendations of DCG-2016.

Our findings also indicated that the total CHEI score was higher in female participants than in male participants, verifying the viewpoint that females have greater quality of diet than males. In this study, female participants exhibited significantly higher component scores for tubers, total vegetables, dark vegetables, fruits, dairy, fish and seafood, and red meats, and male participants only had a significantly higher component score for total grains. An epidemiological study [14] from five universities in the United Kingdom found that female students preferred a ‘vegetarian’ diet including soybeans, nuts, and vegetables, whereas male students preferred a ‘convenience, red meat, and alcohol’ diet including red meat, fried food, and processed meat. Another study from Lazzeri et al. also determined that, regarding vegetable and fruit consumption, female students reported a higher consumption frequency than male students in their food frequency questionnaire [48]. This may be attributed to the fact that female students were more interested in diet, nutrition, and weight [49], whereas male students were disinterested in nutritional advice and health-enhancing activities [50], resulting in the nutritional knowledge level of female students being higher than that of male students [51]. However, it is worth noting that a higher proportion of female students than male students acquired zero points for the CHEI component of added sugars (22.09% versus 13.51%, *p* = 0.142), suggesting that female students may consume more snacks than male students, which requires further attention.

In addition to sex, potential influencing factors of education, dietary health literacy, and time spent sedentarily significantly affected the dietary quality of college students in the medical school. Graves et al. [52] previously reported that graduate students showed significantly higher ratings of healthy eating than undergraduate students, with the proportion of students satisfying the recommended daily servings of vegetables and fruits being higher in graduate students. There was consensus in our study that graduate students had higher total CHEI scores than undergraduate students. Additionally, dietary health literacy reflects an individual’s ability to acquire, comprehend, and apply essential dietary health information and make correct nutrition decisions [29]. A growing body of evidence indicates that college students with lower dietary health literacy have poorer diets and health [53]. Our findings support this observation that the application capacity of the Nutritional Literacy Assessment Questionnaire was positively associated with CHEI scores. Furthermore, students who spend more time sedentarily displayed poorer dietary quality than those who spend less time sedentarily. Currently, increased screen time accounted for the majority of time spent sedentarily in teenagers, including watching TV and playing on the computer [54,55], which may lead to poor food choices and unhealthy dietary habits [56,57].

This study employed the IOS to evaluate the dietary quality of college students, with the medical school of Fudan University being the first school to use the IOS, which began in 2017 in China. Based on the dietary quality of study participants evaluated by the IOS, we found the results of this study seem to be coherent with those of the China Health and Nutrition Survey for adults [58], which adopted the dietary investigation method regarded as a gold standard—multiple consecutive 24-h dietary recalls [59]. It verified that the dietary quality evaluated by the IOS was relatively precise. Compared with food frequency questionnaires and 24-h dietary recalls, use of the IOS enables a large volume of dietary data to be collected in a short amount of time. Moreover, the IOS provides an excellent opportunity to acquire continuous dietary data, evaluate relatively precise dietary quality in the school canteens, and provide appropriate dietary suggestions for students and teachers. In response to the sustainable development goals in China, future dietary assessment surveys based on the IOS can not only provide more efficient and extensive dietary assessment, but also have great potential for reducing the consumption of human, material, and financial resources. However, there were some limitations existing in the IOS. For example, we needed to reweigh the raw materials and the quantity in grams of each dish when the dishes supplied by school canteen were updated. Thus, it is difficult to keep the dishes database up to date. Meanwhile, we observed that college students at the medical school consumed a large quantity of foods outside the school canteen, which may have affected the assessment of dietary quality. Thus, in order to precisely evaluate the dietary quality and extend the system to more schools, collective units, and communities, it is essential to verify the reliability and validity of the IOS in future studies.

## 5. Strengths and Limitations

There are several strengths in this study. Firstly, the meal data for the school canteen from the IOS were consecutive, relatively objective, and accurate. Our study was the first to apply one-year canteen data combined with CHEI scores to relatively precisely evaluate dietary quality in 283 male and female students at the medical school, providing the opportunity to monitor students’ diets continuously. Secondly, this study explored the potential factors influencing the dietary quality of college students in the medical school and provided a possible pathway for improving dietary quality in the future. Several limitations were also present in this study. Firstly, the sample size of study participants was relatively small, requiring the need to expand the sample size for further analysis in the future. Secondly, the time of acquiring data was different between the canteen data from the IOS and the questionnaire survey, which may weaken the accuracy of outcomes. However, we designed the question regarding the changes in participants’ diets in the past two years to decrease this bias. In fact, we evaluated the dietary intake of the SFFQ and whether the dietary habits of the last two years had changed to represent the supplementary dietary intake outside the school canteens during the IOS data collection period. This is a major limitation of this study, which needs to be further explored. Thirdly, because the IOS has not achieved wide application, the replication of the results obtained in this study may be limited.

## 6. Conclusions

Based on relatively objective dietary data from the IOS and SFFQ, our research revealed that the overall dietary quality of college students in the medical school in Shanghai, China, needs to be further improved, with low intakes of total grains, fruits, soybeans, fish and seafood, and seeds and nuts, and high intakes of added sugars. Compared with males, females had better diet quality and conformed more strongly with the recommendations of DCG-2016, but it is worth noting that there was a higher proportion of female students consuming added sugars than male students. Meanwhile, school canteens need to provide students with sufficient fish and seafood on the premise of ensuring food safety. Potential influencing factors, including education, dietary health literacy, and amount of time spent sedentarily, should be noted to improve the dietary quality of college students on a day-to-day basis.

## Figures and Tables

**Table 1:** The demographic characteristics of male and female college students in the medical school (*n* = 283).

Characteristics		Male (*n* (%))	Female (*n* (%))	*χ^2^* Value	*p* Value
Age (years)	<26	72 (64.90)	106 (61.60)	0.303	0.582
	≥26	39 (35.10)	66 (38.40)		
Education	Undergraduate	64 (57.70)	62 (36.00)	12.756	<0.001
	Graduate	47 (42.30)	110 (64.00)		
Major	Medical major	107 (96.40)	159 (92.40)	1.869	0.172
	Medical-related major	4 (3.60)	13 (7.60)		
Household type	Urban	53 (47.70)	96 (55.80)	1.761	0.185
	Countryside	58 (52.30)	76 (44.20)		
Resident student	Yes	106 (95.50)	169 (98.30)	1.871	0.171
	No	5 (4.50)	3 (1.70)		
Dietary habit	General diet	101 (91.00)	169 (98.30)	8.124	0.004
	Other diet ^a^	10 (9.00)	3 (1.70)		
BMI (kg/m^2^)	Underweight	3 (2.70)	37 (21.50)	47.435	<0.001
	Normal weight	73 (65.80)	126 (73.30)		
	Overweight and obesity	35 (31.50)	9 (5.20)		
Smoking	Yes	4 (3.60)	0 (0.00)	6.287	0.012
	No	107 (96.40)	172 (100.00)		
		**Median (IQR)**	**Median (IQR)**	***Z* value**	
Nutrition Literacy Assessment Questionnaire	Acquisition capacity	12.00 (8.00, 13.00)	12.00 (11.00, 15.00)	−3.942	<0.001
	Comprehension capacity	22.00 (18.00, 24.00)	23.00 (20.00, 24.00)	−1.900	0.058
	Application capacity	10.00 (9.00, 12.00)	12.00 (9.00, 12.00)	−3.700	<0.001
Sedentary time (hour/day)	—	8.00 (5.00, 10.00)	8.00 (6.00, 10.00)	−0.680	0.497
Leftovers rate (%)	—	10.00 (5.00, 10.00)	10.00 (10.00, 20.00)	−4.434	<0.001

Abbreviation: BMI, Body Mass Index; IQR, inter-quartile range. ^a^ other diet included Muslim and vegetarian diets. Chi-square test and Wilcoxon rank sum test were applied in an equilibrium test between demographic characteristics of males and females.

**Table 2:** Comparison of CHEI and its component scores between males and females.

CHEI and Component Scores	Total Participants	Male (*n* = 111)	Female (*n* = 172)	*p* Value
CHEI	66.65 (63.17, 70.41)	64.31 (61.31, 68.59)	68.38 (64.63, 71.95)	<0.001
Total Grains	2.95 (2.57, 3.33)	3.25 (2.79, 3.64)	2.78 (2.40, 3.14)	<0.001
Whole Grains and Mixed Beans	5.00 (4.87, 5.00)	5.00 (4.84, 5.00)	5.00 (4.87, 5.00)	0.588
Tubers	3.85 (2.85, 5.00)	3.54 (2.60, 4.90)	4.13 (2.99, 5.00)	0.043
Total Vegetables	3.16 (2.66, 3.72)	2.85 (2.51, 3.35)	3.36 (2.85, 3.88)	<0.001
Dark Vegetables	3.35 (2.67, 4.08)	2.95 (2.44, 3.70)	3.59 (2.92, 4.26)	<0.001
Fruits	3.5 (1.94, 5.96)	2.43 (1.16, 3.93)	4.39 (2.71, 6.81)	<0.001
Dairy	3.64 (1.65, 5.00)	2.35 (0.96, 4.58)	4.19 (2.24, 5.00)	<0.001
Soybeans	1.69 (1.30, 2.06)	1.62 (1.30, 2.00)	1.73 (1.35, 2.09)	0.281
Fish and Seafood	1.22 (0.62, 1.94)	0.85 (0.41, 1.76)	1.56 (0.79, 2.27)	<0.001
Poultry	5.00 (5.00, 5.00)	5.00 (5.00, 5.00)	5.00 (5.00, 5.00)	0.143
Eggs	5.00 (5.00, 5.00)	5.00 (5.00, 5.00)	5.00 (5.00, 5.00)	0.516
Seeds and Nuts	1.65 (0.48, 3.99)	1.41 (0.45, 3.79)	1.72 (0.52, 4.33)	0.692
Red Meats	3.09 (2.65, 3.53)	2.89 (2.41, 3.39)	3.18 (2.85, 3.58)	<0.001
Cooking Oils	7.12 (5.29, 8.67)	7.22 (5.72, 8.58)	6.91 (5.11, 8.71)	0.432
Sodium	7.64 (7.17, 8.34)	7.80 (7.35, 8.49)	7.53 (7.12, 8.33)	0.104
Added Sugars	5.00 (1.47, 5.00)	5.00 (1.87, 5.00)	4.89 (1.22, 5.00)	0.084
Alcohol	5.00 (5.00, 5.00)	5.00 (5.00, 5.00)	5.00 (5.00, 5.00)	0.999

The values in the table are expressed as median (inter-quartile range). The Wilcoxon rank sum test was used to compare the differences of the CHEI and its component scores in male and female participants.

**Table 3:** Analysis of the potential influencing factors of the CHEI score by linear regression model.

Characteristics		Univariate Linear Regression Model		Multiple Linear Regression Model ^b^	
		*β* (95 % CI)	*p* Value	*β* (95 % CI)	*p* Value
Age (years)	≥26	1.01 (−0.47, 2.49)	0.179	0.17 (−1.51, 1.85)	0.844
	<26	1.00 (reference)		1.00 (reference)	
Sex	Female	3.77 (2.47, 5.06)	<0.001	2.80 (1.24, 4.35)	<0.001
	Male	1.00 (reference)		1.00 (reference)	
Education	Graduate	2.04 (0.72, 3.36)	<0.001	1.56 (0.23, 2.89)	0.022
	Undergraduate	1.00 (reference)		1.00 (reference)	
Major	Medical-related major	1.33 (−1.48, 4.14)	0.353	−0.06 (−2.79, 2.68)	0.969
	Medical major	1.00 (reference)		1.00 (reference)	
Household type	Countryside	0.13 (−1.21, 1.47)	0.849	0.43 (−0.86, 1.72)	0.509
	Urban	1.00 (reference)		1.00 (reference)	
Resident student	No	−3.06 (−7.08, 0.96)	0.136	−2.56 (−6.47, 1.34)	0.198
	Yes	1.00 (reference)		1.00 (reference)	
Dietary habit	Other diet ^a^	0.28 (−2.92, 3.47)	0.863	2.37 (−0.73, 5.46)	0.133
	General diet	1.00 (reference)		1.00 (reference)	
Nutrition Literacy Assessment Questionnaire	Acquisition capacity	0.23 (0.04, 0.42)	0.018	0.10 (−0.09, 0.29)	0.296
	Comprehension capacity	0.04 (−0.14, 0.21)	0.680	−0.12 (−0.31, 0.08)	0.237
	Application capacity	0.37 (0.11, 0.64)	0.006	0.34 (0.03, 0.66)	0.031
BMI (kg/m^2^)	Underweight	0.95 (−0.98, 2.87)	0.334	0.22 (−1.68, 2.12)	0.819
	Overweight and obesity	−2.47 (−4.32, −0.62)	0.009	−0.99 (−2.86, 0.88)	0.298
	Normal weight	1.00 (reference)		1.00 (reference)	
Smoking	No	4.91 (−0.72,10.55)	0.087	3.01 (−2.46, 8.49)	0.279
	Yes	1.00 (reference)		1.00 (reference)	
Sedentary time (hour/day)		−0.14 (−0.27, −0.01)	0.043	−0.16 (−0.28, −0.03)	0.016

Abbreviation: BMI, Body Mass Index. ^a^ other diet included Muslim and vegetarian diets. ^b^ The model was adjusted by age, sex, education, major, household type, resident student, dietary habit, Nutrition Literacy Assessment Questionnaire, BMI, smoking, and sedentary time.

## Data Availability

The data presented in this study are available on request from the corresponding author. The data are not publicly available due to privacy of study participants.

## Supplementary Materials

The following supporting information can be downloaded at: https://www.mdpi.com/article/10.3390/nu14051012/s1, Figure S1: Introduction of “Intelligent Ordering System”; Table S1: Factor analysis for Nutrition Literacy Assessment Questionnaire.
