# Supplementary material for: Evaluation of Dietary Quality Based on Intelligent Ordering System and Chinese Healthy Eating Index in College Students from a Medical School in Shanghai, China
Source: Nutrients. 2022 Feb 27;14(5):1012. doi: 10.3390/nu14051012 (PMC8912503; doi:10.3390/nu14051012)
Supplement: Supplementary file 1 [file nutrients-14-01012-s001.zip › nutrients-1566830-supplementary.pdf]

Supplemental Materials:

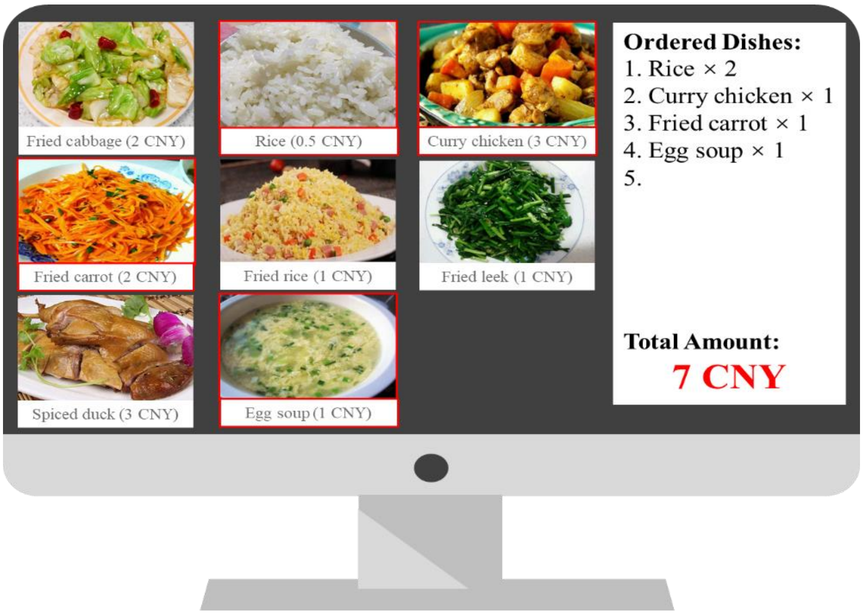

Figure S1. Introduction of "Intelligent Ordering System"

Table S1 Factor analysis for Nutrition Literacy Assessment Questionnaire

	Factor loadings		
	1	2	3
I am vulnerable to nutritional dietary advice on new media (wechat, Weibo)	**0.828**	-0.049	0.031
If I discuss food with others, I often refer to the information in the media	**0.779**	-0.086	0.007
I mainly learn nutrition and diet knowledge from new media (wechat and weibo)	**0.713**	-0.079	0.028
I believe in the various dietary suggestions I read in the media	**0.697**	0.257	-0.154
I think the dietary guidelines are easy to understand	0.024	**0.800**	0.070
I understand the concept of "balanced diet"	0.128	**0.739**	-0.014
I can understand the information on food labels (nutrients and energy, etc.)	0.074	**0.730**	0.115
When I read about nutrition and diet, I don't need someone to help me understand it	-0.190	**0.705**	-0.119
I found the nutritionist's statement easy to understand	-0.169	**0.680**	-0.085
I understand the core items and standards in the dietary guidelines	0.106	**0.633**	0.090
I refer to the label information on the food package (nutrients, energy, etc.) when choosing food	0.001	0.055	**0.811**
I can choose food according to my own nutritional status (such as fat free, less sugar, etc.)	-0.046	0.067	**0.795**
I'm willing to spend extra time or money on healthy meals	-0.023	-0.067	**0.763**
Eigen value	2.604	3.495	1.407
Variance explained (%)	20.028	26.883	10.822
Cumulative sums of squares (%)	46.911	26.883	57.733
